# Infarct zone viability in stable patients with ST-elevation myocardial infarction not undergoing reperfusion - the COAT trial registry

**DOI:** 10.1186/1532-429X-13-S1-P155

**Published:** 2011-02-02

**Authors:** Lukasz A Malek, Mariusz Kruk, Mariusz Klopotowski, Cezary Kepka, Jerzy Rekosz, Irmina Fidala, Zbigniew Binio, Wojciech Krzyżanowski, Witold Ruzyllo, Adam Witkowski

**Affiliations:** 1 Institute of Cardiology, Warsaw, Poland; 2 „MEDITRANS”, Warsaw, Poland; 3 Mazowieckie Centrum Leczenia Chorób Płuc i Gru?licy, Otwock, Poland; 4 Samodzielny Publiczny Zakład Opieki Zdrowotnej, Grojec, Poland; 5 Szpital Powiatowy im. Marii Skłodowskiej-Curie, Ostrow Mazowiecka, Poland

## Background

Occluded Artery Trial (OAT) demonstrated that percutaneous coronary intervention (PCI) with optimal medical therapy does not reduce the frequency of major adverse events compared to optimal medical therapy alone when performed on days 3-28 post ST-elevation myocardial infarction (STEMI) in stable patients. However the assessment of infarct zone (IZ) viability was not used as an inclusion criterion in the this trial. Several studies in patients with stable coronary artery disease have shown that only patients with preserved myocardial viability benefit from revascularization.

## Objective

Therefore we decided to assess the frequency of IZ viability in patients fulfilling the criteria for randomization in the OAT trial.

## Methods

Until now the registry included 16 patients (age 41-77 yrs, 11 male) screened for participation in the Cardiac magnetic resonance for the Occluded infarct-related Artery Treatment (COAT) trial (Clinicaltrials.gov NCT00968383) which is a single center randomized study conducted in the Institute of Cardiology in Warsaw to compare late revascularization with optimal medical therapy alone on days 3-28 post MI in stable patients with preserved IZ viability. Estimated enrollment into the randomized phase is 40 patients. Viability is defined as necrosis transmurality <50% in at least 4 segments of IZ on cardiac magnetic resonance. The primary end-point of the study is a change in systolic wall thickening (SWT) at 6 months. Secondary end-points include change in left ventricular ejection fraction (LVEF), wall motion score index (WMSI), end-diastolic and end-systolic left ventricular volumes (LVEDV, LVESV).

## Results

In the studied group 8 patients (50%) did not have preserved viability of the infarct zone. Patients with preserved IZ viability were randomized to late revascularization (4 patients) or optimal therapy alone (4 patients).

## Conclusions

Lack of preserved IZ viability in 50% of patients fulfilling the criteria for randomization in the OAT trial might have influenced the negative results of this trial regarding late revascularization in STEMI. Hypothesis tested in the ongoing COAT trial assumes that late opening of the occluded infarct-related artery only in patients with preserved myocardial tissue viability may lead to improvement of left ventricular function and/or volumes.

